# Special Issue “Ring-Opening Polymerization”

**DOI:** 10.3390/molecules21121720

**Published:** 2016-12-14

**Authors:** Takeshi Endo, Atsushi Sudo

**Affiliations:** 1 Molecular Engineering Institute, Kinki University, 11-6 Kayanomori, Iizuka, Fukuoka 820-8555, Japan; 2 Department of Applied Chemistry, Faculty of Science and Engineering, Kinki University, Kowakae 3-4-1, Higashi-Osaka, Osaka 577-8502, Japan; asudo@apch.kindai.ac.jp

Ring-opening polymerization (ROP) has gained much attention because of its usefulness to synthesize polymers with main chains inheriting the heteroatoms and functional groups from the corresponding monomers. The functional groups incorporated into the polymers give rise to the polarities of the polymers, by which the polymers exhibit various inherent properties. The ROPs of epoxides, lactones, and lactams are efficient approaches to polyethers, polyesters, and polyamides, which are important classes of polymers with a wide range of applications, and such practical aspects as well as the scientific interest in ROPs have motivated researchers to develop new catalysts, new initiation systems, and new heterocyclic monomers.

In this Special Issue, nine original research articles and two reviews are contributed by prominent researchers. By their efforts, the forefront of the field of ROP that covers (1) new applications of ROP to the synthesis of functional materials; (2) new catalysts; (3) new ROP systems; and (4) new cyclic monomers is clearly presented to us.

Zhang et al. [1] studied the functionalization of xylan—a naturally occurring polysaccharide—by ring-opening graft polymerization of propylene carbonate, which resulted in the significant improvement of the mechanical properties of xylan. They report the use of 1,8-diazabicyclo[5.4.0]undec-7-ene (DBU) as a catalyst, and that the use of an ionic liquid as a reaction medium allow the successful graft polymerization. Sawdon et al. [2] report the ring-opening polymerization of ε-caprolactone initiated from ganciclovir (GCV), a nucleoside analogue with antiviral activity. The polymerization gave a GCV derivative functionalized with polyester chains. Then, to their chain ends, chitosan was attached to construct amphiphilic copolymers, of which micellar nanoparticles are demonstrated to be potentially applicable to a drug releasing system. Perdih et al. [3] applied the ring-opening polymerization of α-amino acid *N*-carboxyanhydride (NCA) to the synthesis of a poly(l-glutamic acid) derivative modified with glucosamine, of which biomedical applications are of great interest. Therein, the carboxyl functions in the side chains of poly(l-glutamic acid) were condensed with a d-(+)-glucosamine in an aqueous system using 4-(4,6-dimethoxy-1,3,5-triazin-2-yl)-4-methylmorpholinium chloride (DMTMM) as a coupling reagent.

Chen et al. [4] developed a series of effective catalysts for the ring-opening polymerization of l-lactide. The catalysts are zinc complexes, of which ligands are aldimine derivatives synthesized from coumarins. The ROP of l-lactide catalyzed by the zinc catalysts proceeded in a living fashion to give the corresponding poly(l-lactide)s with controlled molecular weights. Żółtowska et al. [5] developed a new catalytic system by combining diethylzinc with the non-toxic phenolic compounds gallic acid and propyl gallate. The catalysts thus prepared are effective in the catalysis of the ring-opening polymerization of ε-caprolactone.

Ochiai et al. [6] report on a unique use of the ring-opening reaction of cyclic dithiocarbonates for the synthesis of organic–sulfur–zinc hybrid materials. The reaction gives the corresponding thiols, and these thiols are condensed with zinc acetate to obtain organic–inorganic hybrid polymers. Rosenthal-Kim et al. [7] clarified the radical ring-opening redox mechanism in the polymerization of 3,6-dioxa-1,8-octanedithiol (DODT) that affords poly(disulfide)s. The polymerization proceeds in a biphasic system composed of triethylamine and diluted aqueous solution of hydrogen peroxide, where triethylamine plays two key roles, as solvent for the monomer and as a phase transfer agent.

Takeichi et al. [8] developed a new bifunctional monomer bearing a benzoxazine ring and a styrenic moiety. The radical polymerization of the styrenic part gives the corresponding polymers bearing the benzoxazine rings in the side chains. Upon heating the polymers, the pendent benzoxazine rings undergo ring-opening polymerization to afford crosslinked polymers. Some of them are thermally stable and tough, and are thus promising as high performance materials. Heiny et al. [9] developed epoxide-type monomers and demonstrated their copolymerizations with l-lactide. The monomers have an ester and protected amino functions, allowing their copolymerizations to be a convenient and efficient method for the synthesis of poly(l-lactide)s bearing functional moieties that can be further modified.

Lalevée et al. [10] have reviewed the recent progress in the field of photoinitiators (PI) and photoinitiating systems (PIS) for cationic ring-opening polymerization triggered by photo irradiation, citing 119 relevant references. Piotrowska et al. [11] have reviewed enzymatic ring-opening polymerizations using ionic liquids as polymerization media, which is gaining great interest as a green and efficient approach to polyesters. In this review, 135 relevant references are cited.

In conclusion, the research area of ring-opening polymerization (ROP) is growing at an unprecedented rate, finding a wide range of applications. As editors of this Special Issue, we thank all of the authors for their contributions and the staff of MDPI for their editorial support.
